# Redox properties and human serum albumin binding of nitro-oleic acid

**DOI:** 10.1016/j.redox.2019.101213

**Published:** 2019-05-08

**Authors:** Martina Zatloukalova, Milos Mojovic, Aleksandra Pavicevic, Martin Kabelac, Bruce A. Freeman, Michaela Pekarova, Jan Vacek

**Affiliations:** a Department of Medical Chemistry and Biochemistry, Faculty of Medicine and Dentistry, Palacky University, Hnevotinska 3, Olomouc 775 15, Czech Republic; b Faculty of Physical Chemistry, University of Belgrade, Studentski trg 12-16, Belgrade, Serbia; c Department of Chemistry, Faculty of Science, University of South Bohemia, Branisovska 31, Ceske Budejovice 370 05, Czech Republic; d Department of Pharmacology and Chemical Biology, University of Pittsburgh School of Medicine, Pittsburgh PA, 15261, USA; e The Czech Academy of Sciences, Institute of Biophysics, Kralovopolska 135, Brno 612 65, Czech Republic

**Keywords:** Electrophiles, Nitrated fatty acids, Oleic acid, NO, Proteins, Serum albumin binding, ACV, alternating-current voltammetry, BSA, bovine serum albumin, CPSA, constant-current chronopotentiometric stripping analysis, CV, cyclic voltammetry, DFT, density functional theory, 16-DS, 16-doxyl stearic acid, EPR, electron paramagnetic resonance, FA, fatty acid, Fe(DTCS)_2_, complex of Fe with N-(dithiocarboxy)sarcosine, GSH, glutathione, HMDE, hanging mercury drop electrode, HOMO, highest occupied molecular orbital, HSA, human serum albumin, LUMO, lowest unoccupied molecular orbital, NO_2_-OA, nitro-oleic acid, OA, oleic acid, PAGE, polyacrylamide gel electrophoresis, PCM, polarizable continuum model, PGE, pyrolytic graphite electrode, PPAR, peroxisome proliferator-activated receptor, RONS, reactive oxygen and nitrogen species, SDS, sodium dodecyl sulfate, SLFA, spin-labeled fatty acid, SNP, sodium-nitroprusside, SWV, square-wave voltammetry

## Abstract

Nitro-fatty acids modulate inflammatory and metabolic stress responses, thus displaying potential as new drug candidates. Herein, we evaluate the redox behavior of nitro-oleic acid (NO_2_-OA) and its ability to bind to the fatty acid transporter human serum albumin (HSA). The nitro group of NO_2_-OA underwent electrochemical reduction at −0.75 V at pH 7.4 in an aqueous milieu. Based on observations of the R–NO_2_ reduction process, the stability and reactivity of NO_2_-OA was measured in comparison to oleic acid (OA) as the negative control. These electrochemically-based results were reinforced by computational quantum mechanical modeling. DFT calculations indicated that both the C9-NO_2_ and C10-NO_2_ positional isomers of NO_2_-OA occurred in two conformers with different internal angles (69° and 110°) between the methyl- and carboxylate termini. Both NO_2_-OA positional isomers have LUMO energies of around −0.7 eV, affirming the electrophilic properties of fatty acid nitroalkenes. In addition, the binding of NO_2_-OA and OA with HSA revealed a molar ratio of ~7:1 [NO_2_-OA]:[HSA]. These binding experiments were performed using both an electrocatalytic approach and electron paramagnetic resonance (EPR) spectroscopy using 16-doxyl stearic acid. Using a Fe(DTCS)_2_ spin-trap, EPR studies also showed that the release of the nitro moiety of NO_2_-OA resulted in the formation of nitric oxide radical. Finally, the interaction of NO_2_-OA with HSA was monitored *via* Tyr and Trp residue electro-oxidation. The results indicate that not only non-covalent binding but also NO_2_-OA-HSA adduction mechanisms should be taken into consideration. This study of the redox properties of NO_2_-OA is applicable to the characterization of other electrophilic mediators of biological and pharmacological relevance.

## Highlights

• Nitro fatty acids (FAs) are characterized as redox reactive electrophiles.
• Special attention is given to 9,10-nitrooleate (NO_2_-OA) redox transformation.
• The binding of NO_2_-OA to the FA transported human serum albumin is described.
• Finally, NO release properties and a stability evaluation of NO_2_-OA are discussed.

## Graphical abstract

**Figure:**
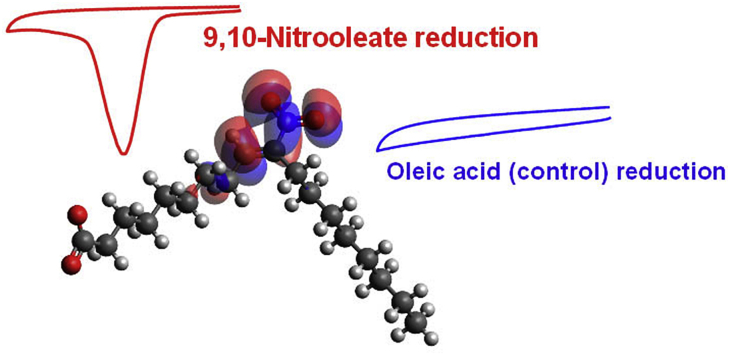

## 1 Introduction

Inflammatory and metabolic reactions increase rates of production and levels of reactive oxygen and nitrogen species (RONS), including nitric oxide and nitrogen dioxide radicals, hydroxyl radicals, hydrogen peroxide, superoxide, and their downstream reaction products [1]. These species also mediate the generation of electrophilic products such as fatty acid nitroalkenes, malondialdehyde and 4-hydroxy-2-nonenal, which accept electrons from electron-rich donor molecules (nucleophiles) such as cysteine, histidine and amine moieties [2]. In general, electrophiles react with nucleophiles to form adducts *via* S_N_1 and S_N_2 substitution, 1,4-addition, Schiff-base formation, and radical-mediated reactions [3,4].

Thiols are the primary nucleophilic targets in biology, due to an intrinsic abundance and reactivity with various electrophiles (including oxidants), therefore are important in modulating signaling, detoxification, and antioxidant responses [5]. Glutathione (GSH) is the main low-molecular-weight thiol in the cytosol [6]. In the blood, electrophiles form adducts with proteins such as human serum albumin (HSA) [4,7]. Albumin is also important for reversible binding and transport of acidic and lipophilic compounds in plasma, including fatty acids (FAs). Reactions of electrophiles with HSA often occur with the Cys34 thiol and amine groups of His, Trp, Lys and the *N*-termini [8]. The adductome of HSA-Cys^34^ affirms that this moiety is the most abundant and reactive nucleophile in serum [8].

Nitro-fatty acids (NO_2_-FAs) are endogenously-occurring electrophiles that are generated through the reaction of RONS with unsaturated fatty acids. The mechanisms of NO_2_-FAs formation primarily center on the reactions of NO_2_, generated by peroxynitrite (ONOO^−^), the oxidation of nitrite (NO_2_^−^) by heme peroxidases and the protonation of NO_2_^−^, a reaction that yields nitrous acid (HNO_2_) and downstream nitrating and nitrosating species [9]. A significant increase in the levels of NO_2_-FAs occurs during various pathological conditions such as ischemia reperfusion injury and in response to mediators produced by viral and bacterial infections [10,11]. There are also several mechanisms that inactivate NO_2_-FA signaling as well, including reduction of the alkene and the export of thiol-NO_2_-FA adducts by the multi-drug resistance protein family [[12], [13], [14]].

NO_2_-FAs induce multiple pleiotropic signaling responses, resulting in anti-inflammatory and antioxidant effects [9]. These actions have been attributed to the electrophilic capacity of the β-carbon of the reactive nitroalkenyl substituent of NO_2_-FAs, which undergo a kinetically rapid and reversible Michael addition [15]. This reaction, termed nitroalkylation, occurs primarily with functionally-significant Cys residues of transcription factors and enzymes [15,16]. Only a residual amount of nitro-oleic acid (NO_2_-OA) is directly detectable in the vascular compartment in the “free form”. The remaining pool of NO_2_-OA (over 90%) is reversibly bound to plasma and tissue thiols (*e.g.* cysteine and its metabolites, GSH and serum proteins) *via* Michael addition [17].

In transducing its signaling actions, NO_2_-OA and other fatty acid nitroalkenes react with susceptible nucleophilic amino acid residues to post-translationally modify proteins. For example, the reversible nitroalkylation of red cell glyceraldehyde-3-phosphate dehydrogenase was first observed [18]. The electrophilic nature of NO_2_-OA also results in the alkylation of recombinant NF-kappaB p65 [19]. Moreover, NO_2_-OA is a non-competitive inhibitor of xanthine oxidoreductase [20] and 5-lipoxygenase [21] and is a partial agonist of peroxisome proliferator-activated receptor-γ [22]. Of functional significance in blood pressure regulation, NO_2_-OA nitroalkylates angiotensin I receptor and soluble epoxide hydrolase [23]. Finally, NO_2_-OA modifies Keap1 to activate gene transcription by nuclear factor E2-related factor-2 [24].

In contrast to protein nitroalkylation, NO_2_-FAs have been suggested to mediate NO release [[25], [26], [27]] *via* a modified Nef reaction of the nitro moiety [[27], [28], [29]]. The release of NO from NO_2_-FAs is a very minor or negligible component of their biological actions since an acute injection does not impact blood pressure or heart rate and the β-carbon adjacent to the nitro group is strongly electrophilic to preferentially react with proteins and thiols [18,30,31]. Of note, NO release from NO_2_-linoleic acid has been proposed to mediate the *S*-nitrosylation of the pro-inflammatory member CD40 to cause subsequent inactivation, thereby triggering an anti-inflammatory response [32]. Therefore, one cannot completely rule out that this NO release can have important consequences for anatomically distinct cellular targets [33], a precept that motivates the present studies.

**Fig. 1:**
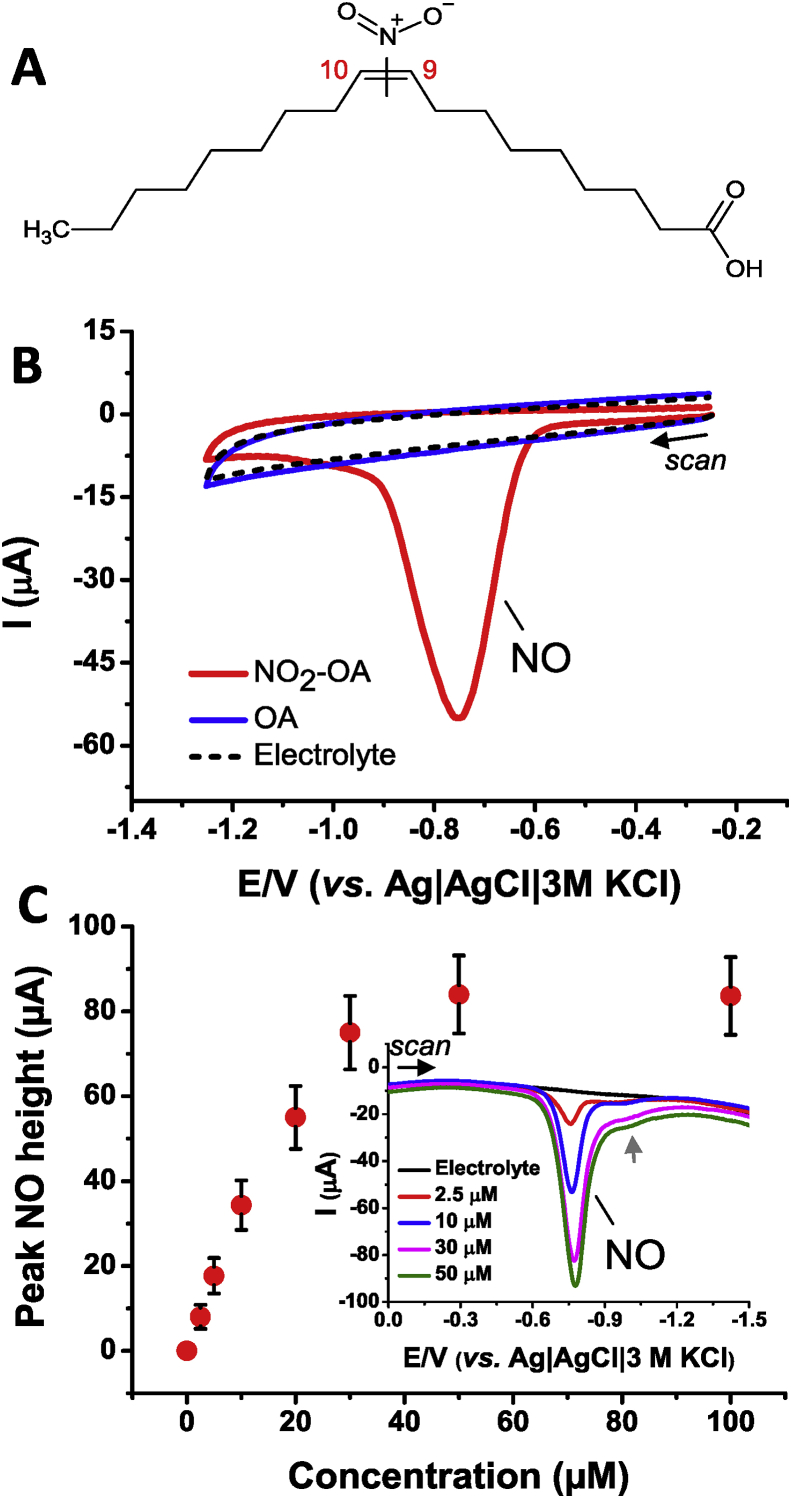
**(A)** Structure of nitro-oleic acid, NO_2_-OA. Electrochemistry of NO_2_-OA on pyrolytic graphite electrode: **(B)** Cyclic voltammograms of 20 μM NO_2_-OA and oleic acid (OA) in 0.1 M phosphate buffer at pH 7.4. CV conditions: start potential −0.25 V, vertex potential −1.25 V, step potential 5 mV, scan rate 1 V/s. **(C)** Dependence of SWV peak NO (*E*_p_ = −0.75 V) on concentration of NO_2_-OA in Britton-Robinson buffer at pH 7.4. SWV parameters: initial potential 0 V, end potential −1.5 V, step potential 5 mV, amplitude 25 mV, frequency 200 Hz. *Inset*: Selected SWV records related to panel **C**.

More detailed mechanistic studies of electrophiles and their interaction with proteins necessitates the use of methods that are capable of detecting, identifying and characterizing both protein-electrophile adducts and non-covalent interactions. Here we utilized electrochemical and EPR approaches to better understand NO_2_-OA reactivity and protein binding properties. Herein we (a) evaluate the stability and reactivity of NO_2_-OA (Fig. 1A) in an aqueous environment at various pH, (b) investigate the binding capacity of NO_2_-OA to human serum albumin (HSA), (c) perform quantum mechanical modeling for a more in-depth perspective of the structural and electronic properties of NO_2_-OA, and (d) apply EPR techniques to evaluate the potential for nitro-moiety release (nitric oxide radical formation) and binding affinity to HSA. This experimental and theoretical work utilizes oleic acid (OA) as a negative control.

## 2 Experimental

### 2.1 Chemicals

Chemicals were purchased from Sigma Aldrich (St. Louis, MO, USA) or BioRad Laboratories (Hercules, CA, USA). 9-Nitrooleate and 10-nitrooleate as pure positional isomers were from Cayman Chemical (Ann Arbor, MI, USA); the purity of both was higher than 98%. NO_2_-OA as an equimolar mixture of the 9- and 10- positional isomers was provided by Bruce Freeman's lab (University of Pittsburgh) in a purity of >98%; methanolic stock solutions were prepared. Unless stated otherwise, NO_2_-OA was used throughout the study as an appropriate mixture of both positional isomers; only the experiment in Fig. S3 (panel B) was performed with pure 9- or 10-nitro isomers. Fatty acid-free HSA was purchased from Sigma Aldrich (no. A3782) at a purity >99%. All solutions were prepared using Milli-Q water (18.2 MΩ cm^−1^), Millipore, Bedford, MA, USA.

### 2.2 Electrochemistry

All electrochemical measurements were performed at room temperature with a μAutolab III analyzer (EcoChemie, Utrecht, Netherlands) in a three-electrode setup with an Ag|AgCl|3 M KCl electrode as the reference and platinum wire as the auxiliary electrode. Two types of working electrodes were used: HMDE (hanging mercury drop electrode; area 0.4 mm^2^) for constant-current chronopotentiometric stripping (CPS) analysis and alternating-current voltammetry (ACV), and a basal-plane pyrolytic graphite electrode (PGE, area 9 mm^2^, source of PG: Momentive Performance Materials, USA) for cyclic and square-wave voltammetry (CV and SWV). Individual settings for electrochemical experiments, as well as concentrations of the compounds, are given in the Figure legends.

The electrochemical analyses were performed *in situ* using the following supporting electrolytes: 0.1 M phosphate and Britton-Robinson buffers, with the exception of SWV studies on the NO_2_-OA interaction with HSA where *ex situ* (adsorptive transfer stripping) analyses were performed using 0.1 M acetate buffer. For the voltammetry of NO_2_-OA, all electrolytes were deaereated using an argon stream. Deaereation was not performed for the interaction studies with HSA, because oxygen does not interfere with CPSA.

The pH measurements were carried out with a HI 2211 pH/ORP Meter (HANNA instruments, IT).

### 2.3 Stability of NO_2_-OA

The stability of 8 μM NO_2_-OA was investigated directly using CPSA in phosphate or Britton-Robinson buffer (supporting electrolyte) at pH 5; 7.4 and 9 for at least 24 h in the presence of atmospheric oxygen. As a control, freshly prepared methanolic solution of 30 mM NO_2_-OA was diluted to 8 μM in the supporting electrolyte for CPSA for each time interval.

### 2.4 Interaction of NO_2_-OA with HSA

The interaction of FAs with HSA (constant concentration 6.25 μM) was studied in 0.1 M phosphate buffer (pH 7.4) at 37 °C. The concentration of FAs was different depending on the molar ratios: 1:1, 2:1, 4:1, 8:1, 16:1, 32:1, and 64:1 [FAs]:[HSA]. After the incubation period, an appropriate volume of incubation mixture was transferred into an electrochemical cell and CPS analysis was performed in 0.1 M phosphate buffer (pH 6.5) at a final HSA concentration of 500 nM. For SWV monitoring of the interactions of FAs with HSA, after 24 h (or 48 h) of incubation the incubation mixtures with 6.25 μM of HSA, containing FAs in appropriate molar ratios, were directly adsorbed onto the electrode surface using an *ex situ* adsorptive pre-concentration procedure [34]. After a 30 s accumulation period, the electrode was washed with deionized water and SWV was performed with a modified electrode in 0.1 M acetate buffer at pH 5.

### 2.5 Gel electrophoresis

Albumin was characterized by sodium dodecyl sulfate-polyacrylamide gel electrophoresis (SDS-PAGE) and by non-denaturing (native) polyacrylamide gel electrophoresis (PAGE) using the 4–15% Mini-Protean^®^ TGX™ gels (BioRad) according to Laemmli [35]. The albumin samples were mixed 1:4 with sample buffer (125 mM Tris–Cl, pH 6.8, 4% (w/v) SDS, 20% (v/v) glycerol, 200 mM dithiothreitol, 0.02% (w/v) bromophenol blue) and heated for 5 min at 95 °C. The samples (5 μg of protein per lane) were subjected to electrophoresis using an electrode buffer containing 25 mM Tris, 192 mM glycine, 0.1% (w/v) SDS, pH 8.3. The gels were subsequently incubated for 15 min in staining solution containing 0.1% (w/v) Coomassie brilliant blue G-250, 40% (v/v) methanol, 10% (v/v) glacial acetic acid, and 50% (v/v) water. The gels were destained in 40% (v/v) methanol, 10% (v/v) glacial acetic acid, 50% (v/v) water until the background became clear.

The non-denaturing PAGE employed a system developed by Ornstein [36] and Davis [37]. The protein samples were mixed 1:4 with sample buffer (125 mM Tris–Cl, pH 6.8, 20% (v/v) glycerol, 0.02% (w/v) bromophenol blue) and subjected to electrophoresis (5 μg of protein per lane) using a buffer consisting of 25 mM Tris, 192 mM glycine, pH 8.3. Proteins in the gels were visualized by Coomassie blue staining as described above.

Thermo Scientific Protein Ladder for SDS-PAGE and SERVA Protein Marker for native PAGE were used as molecular weight markers.

### 2.6 EPR spectroscopy

#### 2.6.1 Interaction of NO_2_-OA with HSA studied by spin-probing method

HSA was dissolved in 100 mM phosphate buffer, pH 7.4, so that the final concentration of HSA was 0.1 mM. The entire volume of this solution was incubated with 0.6 mM 16-DS (16-doxyl stearic acid) until the maximum binding of 16-DS was achieved. The methanolic solutions of NO_2_-OA or OA were added to the bottom of the empty test tube, and the methanol was evaporated in a vacuum concentrator. The amounts of NO_2_-OA and OA were such that the [FA]:[HSA] molar ratios were 1:1, 2:1, 4:1, 8:1 and 16:1. Afterwards, the solution of HSA pre-labeled with 16-DS was added to the tubes containing the NO_2_-OA or OA. The samples were gently vortexed, and subsequently incubated for 30 min at 37 °C in a water bath. Afterwards, the samples were cooled to room temperature for 10 min. After that, the HSA/16-DS/FA solutions were withdrawn into gas-permeable Teflon capillary tubes (Zeus Industries, Raritan, USA), and placed in an EPR resonator cavity.

#### 2.6.2 Detection of NO release from NO_2_-OA

In order to study whether NO_2_-OA is able to release NO, the complex of Fe^2+^ with *N*-(dithiocarboxy)sarcosine (Chemos Cz, Czech Republic, Prague), Fe(DTCS)_2_, was used as an NO-trapping agent. For these experiments, all solutions were prepared in water (or 100 mM phosphate buffer, pH 7.4) bubbled with N_2_ for at least 30 min to remove O_2_. A solution of FeSO_4_ × 7H_2_O (final conc. 1.8 mM) was added to a solution of (NH_4_)_2_DTCS (final conc. 3 mM), followed by adding small volume of NO_2_-OA methanol solution (final conc. 25 mM). Afterwards, the final samples were withdrawn into gas-permeable capillary tubes, and placed in the EPR resonator cavity. In order to obtain the reference EPR signal of NO-adduct, sodium-nitroprusside (SNP; final conc. 8.33 mM), an NO donor, was mixed with the same amounts of FeSO_4_ × 7H_2_O and (NH_4_)_2_DTCS.

#### 2.6.3 EPR data acquisition and processing

EPR spectra were recorded using a Bruker Elexsys II E540 EPR X-band spectrometer at 9.85 GHz and 10 mW microwave power. For all measurements, the modulation amplitude was 2 G, modulation frequency 100 kHz, and conversion time 58.59 ms. Sweep width was 100 G and 77 G for the spin-probing and NO-trapping experiments, respectively.

### 2.7 Calculations

The structures of OA and its 9- and 10-nitro derivatives were optimized at the DFT level of theory employing the 6–311++G(d, p) basis set and B3LYP functional. A systematic torsional search around the C-C bonds linked to the C=C bonds was performed to find the most stable conformers. An analysis of frontier molecular orbitals and molecular electrostatic potentials were done using Avogadro software (http://avogadro.cc). The role of the solvent (water, methanol and *n*-octanol) was treated in an implicit way using the PCM model [38]. All quantum mechanical calculations were performed in the program Gaussian16 [39].

### 2.8 HSA structure visualization

The 3D molecular models of HSA were prepared in PyMOL (Molecular Graphics System, v1.7.4.5 Schrodinger, LLC) where the crystal structure of the HSA was taken directly from the Protein Data Bank (1GNI) [40].

## 3 Results and discussion

### 3.1 Redox behavior of NO_2_-OA

The reduction of NO_2_-OA (Fig. 1A) was first investigated using voltammetry at the PGE with a basal-plane configuration. For this purpose, a NO_2_-OA sample containing an equimolar ratio of both 9- and 10-nitro regioisomers was used. The NO_2_-OA and OA, serving as negative control, were pre-solubilized in methanol and subsequently solvated in buffered supporting electrolytes. The residues of the alcohol, usually less than 0.1%, *v*/*v*, did not interfere with the electrochemical analysis. The electrophilic character of NO_2_-OA corresponds to a reduction process (peak NO) that can be observed around the potential of −0.75 *vs*. Ag|AgCl|3 M KCl at pH 7.4 using CV, see Fig. 1B. Reduction of the nitro group is irreversible and potentially analogous to that observed with a wide range of nitroalkenes, for example hydroxymethylated nitroalkenes [41]. Depending on experimental conditions, R–NO_2_ could be reduced to hydroxylamine and eventually to amine derivatives as reviewed in Ref. [42]. In addition, the multi-component reduction process could result in formation of redox-active reduction products or dimers [43], which will need further investigation in NO_2_-OA. OA was not subjected to redox transformation under the experimental conditions used, as indicated in Fig. 1B (blue line). It was also shown that NO_2_-OA reduction is pH-dependent (Fig. S1 in Supporting Information). The potential of the NO peak was shifted towards more negative values with increasing pH, and the maximum of the peak current response can be found at a slightly acidic or neutral pH. These results are useful for monitoring proton-dependent processes and for an analysis of NO_2_-OA stability, see below.

NO_2_-OA was examined in Britton-Robinson or 0.1 M phosphate buffer, in the adsorbed state, as evidenced by CV at different scan rates *v*. The non-linear approach to the peak NO current on *v*^1/2^ indicates that the reduction is accompanied by an adsorption process (Fig. S2, in Supplementary Information) in agreement with CV theory [44]. The adsorption behavior was also confirmed using square-wave voltammetry (SWV). We found that with increasing NO_2_-OA concentration in the electrolyte, the cathodic NO peak height gradually increased to 50 μM (Fig. 1C). At higher concentrations, the surface of the electrode is fully covered with NO_2_-OA, and the reduction peak does not increase further. The gradual coverage of the electrode surface is evident from time dependence studies as well, Fig. S3A (Supplementary Information). In this experiment, the peak NO of 8 μM NO_2_-OA increased in a similar manner up to 10 s with increasing accumulation time, following the Langmuir isotherm, as demonstrated in Fig. 1C. In addition to peak NO, a shoulder peak (post-wave) at around −1.1 V was also observed (Fig. 1C, grey arrow marked), but it was not of analytical significance for this study.

**Fig. 2:**
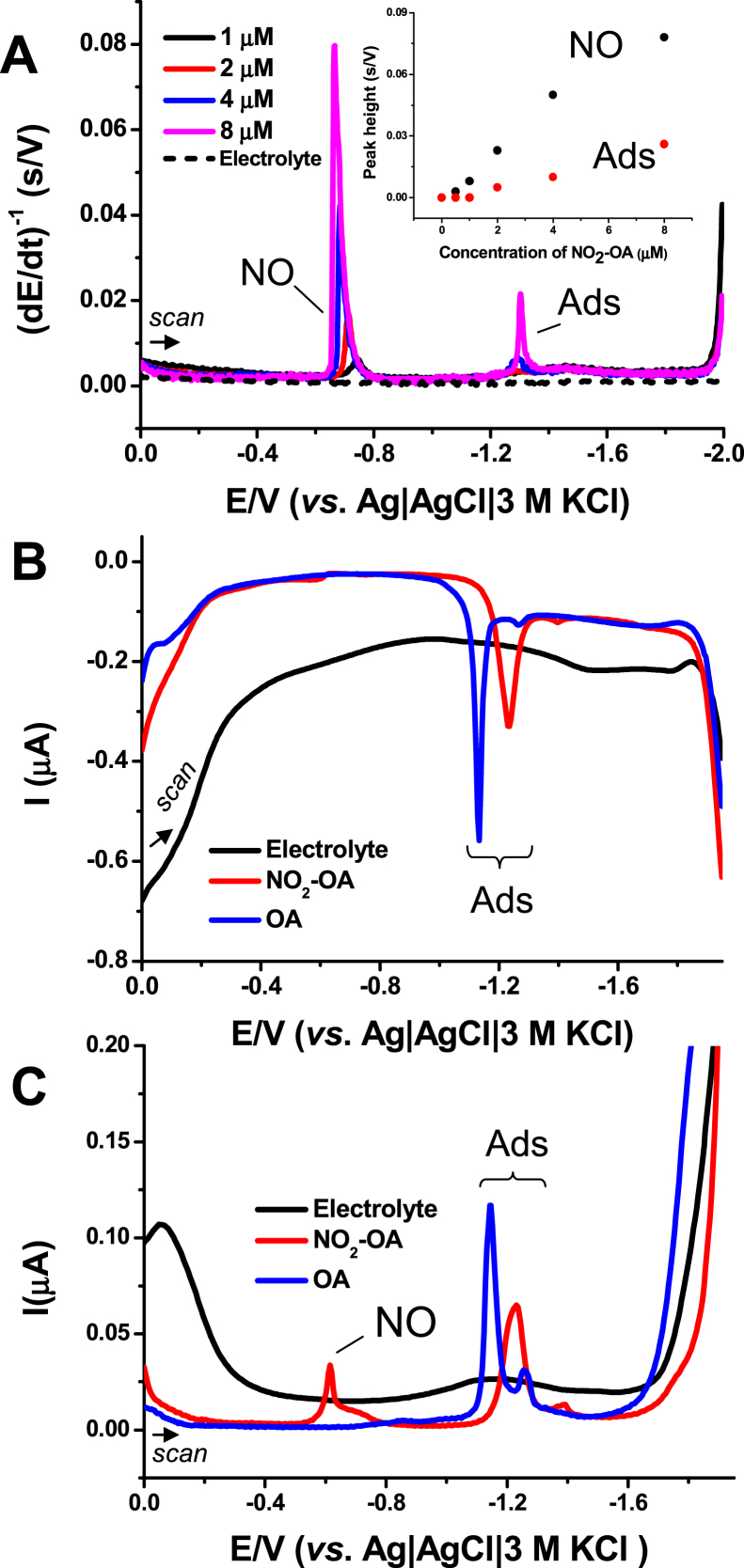
Electrochemistry of NO_2_-OA on mercury electrode, HMDE. **(A)** CPS records of NO_2_-OA at various concentrations in 0.1 M phosphate buffer at pH 7.4; *I*_str_ = −35 μA. *Inset*: Dependence of both CPS peaks NO and Ads on NO_2_-OA concentration. Out-of-phase **(B)** and in-phase **(C)** AC voltammograms of 30 μM NO_2_-OA (red lines) and OA (blue lines) in 0.1 M phosphate buffer (pH 7.4); initial (0 V) and end (−1.95 V) potentials, frequency: 66.2 Hz, amplitude: 5 mM, phase angle: 90° (for **B**) and 0° (for **C**). (For interpretation of the references to colour in this figure legend, the reader is referred to the Web version of this article.)

Then, the electrochemical reduction of NO_2_-OA was studied using constant-current chronopotentiometric stripping analysis (CPSA) at the mercury electrode – HMDE. In addition to the NO reduction peak, another peak at around −1.3 V was observed, see the Ads peak in Fig. 2A. This CPS peak, however, is not related to electron exchange but to the reorientation (adsorption-desorption) of the NO_2_-OA. This was verified by using alternating-current voltammetry (ACV) [45]. Specifically, the out-of-phase ACV, which is sensitive to adsorption-desorption (reorientation) processes, only exhibits a tensametric Ads peak or double peak. This behavior is not only characteristic for NO_2_-OA, but also for OA (Fig. 2B). With in-phase ACV, which recognizes both Faradaic and adsorption-desorption processes, NO and Ads peaks were observed exclusively for NO_2_-OA (Fig. 2C).

All electrochemical experiments were performed with an equimolar mixture of NO_2_-C9 and -C10 positional isomers of OA (Fig. 1A). For a more in-depth perspective of potential isomeric effects, CPS analyses were performed with pure NO_2_-OA positional isomers, as shown in Fig. S3B (Supplementary Information). It is evident that positional isomerism does not affect the general redox behavior of NO_2_-OA. In addition to reduction processes, we also tried to measure the full CV, including the anodic region, see Fig. S1A (Supplementary Information). For this purpose, PGE was first polarized to negative potential values to reduce the NO_2_ group, followed by anodic polarization (Fig. S4 in Supporting Information). This resulted in an anodic peak (*) at around +0.5 V, corresponding to the oxidation of the NO_2_-OA reduction product, confirmed by vertex potential switching. If the vertex potential was adjusted to a less negative potential than the potential of the NO peak, no peak (*) was observed (blue line, Fig. S4). There is no significance of peak (*) for this study, because the NO peak is highly reproducible with a higher current response.

### 3.2 NO_2_-OA structural and electronic properties

The molecular structure and electronic properties of NO_2_-OA were evaluated using computational quantum mechanical modeling based on DFT (density functional theory). The structural interpretations are guided by previous crystal structure and spectroscopic studies of OA [[46], [47], [48]].

**Fig. 3:**
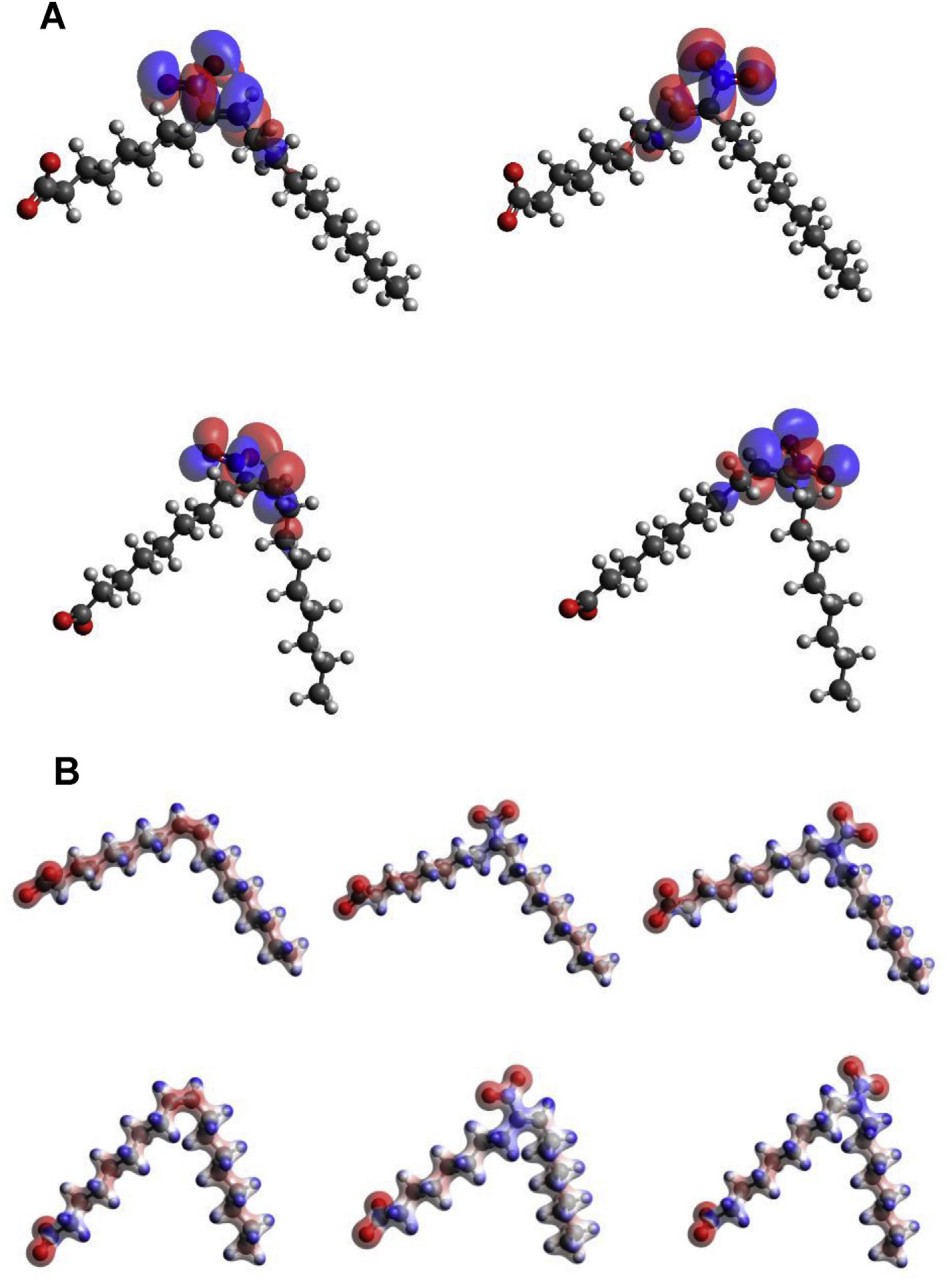
**(A)** Structures with highlighted LUMO for 9-nitrooleic (left) and 10-nitrooleic acid (right) in ‘open’ (top) and ‘closed’ (down) conformations. **(B)** Electrostatic potential visualization for oleic, 9-nitrooleic and 10-nitrooleic acid (from left to right) in ‘open’ (upper) and ‘closed’ (lower) conformations.

Conformational search revealed that there are two stable conformers of OA and its nitro positional isomers which differ in the angle (kink) between the methyl and carboxylate chains (Fig. 3A), resembling the structures obtained by similar DFT calculations [49]. These conformers possess almost the same stability (for unsubstituted OA the difference is 0.8 kJ/mol, for its 9-nitro derivate 0.02 kJ/mol and for its 10-nitroderivative by 1.0 kJ/mol, see Table S1, Supplementary Information). The more stable structure of OA corresponds to the tighter packed (closed) conformation resulting in the angle between the methyl and carboxylate chains being 73°. The presence of NO_2_ group in position C9 as well as at position C10 changed the angle to 69° in both cases. For the second (open) conformer the corresponding angle between methyl and carboxylate chain is 115° for OA and 110° for its nitro derivatives. A change of solvent has no effect on the geometry or on the relative stability of both conformers.

Analysis of the energies of HOMO and LUMO orbitals and the energy gap between them (Table S2, Supplementary Information) revealed that the LUMO orbital is delocalized over the whole molecule, whereas with its nitro derivatives the contribution of the π* orbital of the nitro group and of the C=C bond is dominant (Fig. 3A). The more negative LUMO of nitro derivatives of OA reduces the HOMO-LUMO gap by 2 eV compared to the unsubstituted compound. The HOMO and LUMO energies are insensitive to the position of the nitro group and they are very similar for the ‘open’ and ‘closed’ conformer. The HOMO-LUMO energy gap enlarges gradually upon increasing the polarity of the solvents. This is predominantly caused by the stabilization of LUMO levels in more polar solvents. The LUMO for NO_2_-OA was calculated to be −0.7 eV, in agreement with the electrophilic character of fatty acid nitro-derivatives and corresponding to the LUMO energy estimated experimentally using a different methodology [50].

The molecular electrostatic potential of the FAs is depicted in Fig. 3B; red represents regions with the most negative electrostatic potential, blue represents regions with the most positive one. The presence of an electron-withdrawing NO_2_ group induces a presence of small positive charge in the neighborhood of the C=C bond, whereas in OA those two carbons are slightly negatively charged.

### 3.3 Electrochemical evaluation of NO_2_-OA binding with HSA

**Fig. 4:**
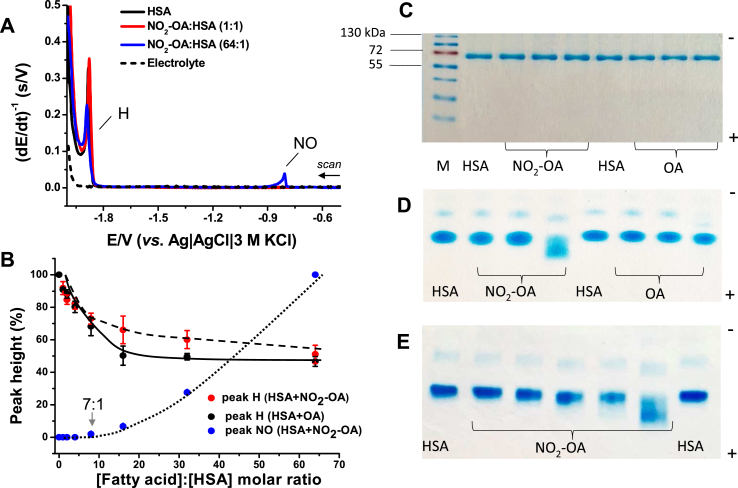
**(A)** CPS records of NO_2_-OA and OA in presence of human serum albumin (HSA). **(B)** Dependence of peak H and NO heights on concentration of NO_2_-OA or OA after their incubation with HSA; *I*_str_ = −95 μA (three independent experiments, *n* = 3). The incubation of HSA with the fatty acids (FAs) was performed for 30 min at 37 °C in 0.1 M phosphate buffer, pH 7.4. Concentration of HSA in incubation mixture was 6.25 μM, and FAs were added in the molar rations: 1:1, 2:1, 4:1, 8:1, 16:1, 32:1, 64:1 [FA]:[HSA]. For CPS analyses, the incubation mixtures were diluted directly in the supporting electrolyte (0.1 M phosphate buffer, pH 6.5) to a final concentration of 500 nM HSA. **(C)** Denaturing SDS and **(D)** native electrophoretograms of HSA in absence or presence of the FAs after 24 h incubation. The samples are in the following order, from left to right: marker (M), HSA, [NO_2_-OA]:[HSA] in molar ratio 4:1, 16:1 and 64:1, HSA, [OA]:[HSA] (4:1, 16:1, 64:1). **(E)** Native electrophoresis of HSA after its 24 h incubation with NO_2_-OA, final [NO_2_-OA]:[HSA] ratio was 4:1, 8:1, 16:1, 32:1, 64:1, from left to right.

**Fig. 5:**
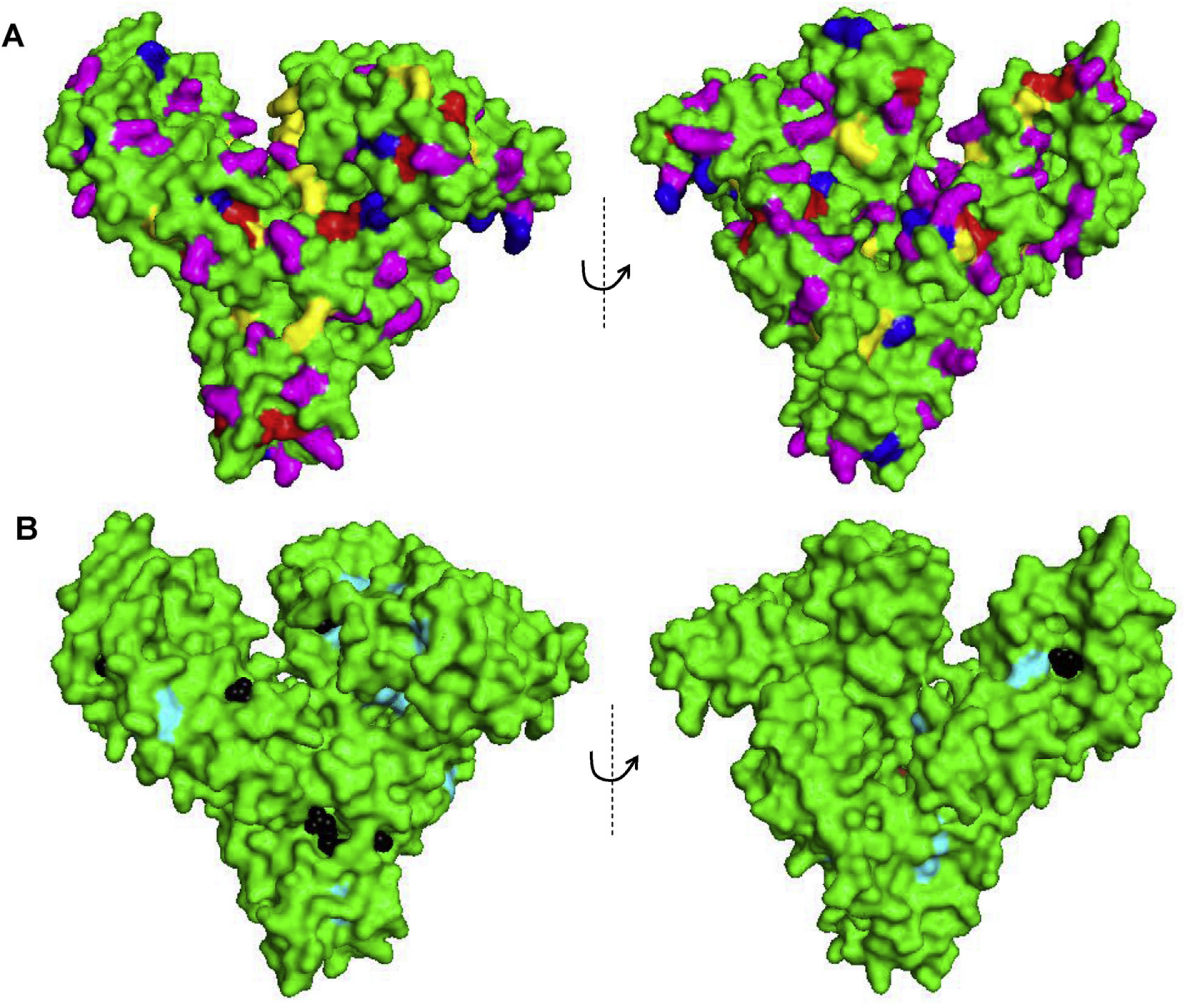
Surface models of HSA (PDB code: 1GNI) with electroactive amino acid residues highlighted. **(A)** Cys – red, His – blue, Arg – yellow, Lys – magenta. **(B)** Tyr – cyan, Trp – brown, oleic acid (OA) – black. The left and right images are mutually rotated by 180° along the vertical axis for each panel. For ribbon models, see Fig. S5 in Supporting Information. (For interpretation of the references to colour in this figure legend, the reader is referred to the Web version of this article.)

For studying the interaction of NO_2_-OA with proteins, fatty-acid-free HSA was interrogated by CPSA at the HMDE. The reason for utilizing CPSA for this purpose is because in a single CPS scan one can not only observe peak NO, corresponding to NO_2_-OA, but also the peak H attributed to HSA (Fig. 4A). Peak H corresponds to the electrocatalytic process in which proton-donating amino acid residues (Cys, His, Lys and Arg) of HSA are involved [51] (Fig. 5A). If the residues are interacting with the binding ligand, they cannot be involved in the electrocatalytic process and thus peak H decreases. This procedure can be applied for evaluating HSA binding capacity, as was recently reported in Refs. [52,53]. In contrast, the cathodic peak of the binding ligand NO_2_-OA is not observable at low concentrations (Fig. 4B), because the ligand binds to HSA and cannot exchange electrons with the electrode. At higher concentrations, when HSA ligand binding sites are saturated, one can observe a gradual increase in peak NO. This effect is attributed to the fact that un-bound NO_2_-OA is present (free) in solution and thus can undergo reduction. The transition point in the curve was observed at a binding stoichiometry of 7:1 [FA]:[HSA] (Fig. 4B, grey arrow marked). The decrease in peak H was observed for both native and nitrated FAs, indicating that the binding capacity for NO_2_-OA and OA could be similar. More details on FA-HSA binding stoichiometry can be found in EPR section bellow.

**Fig. 6:**
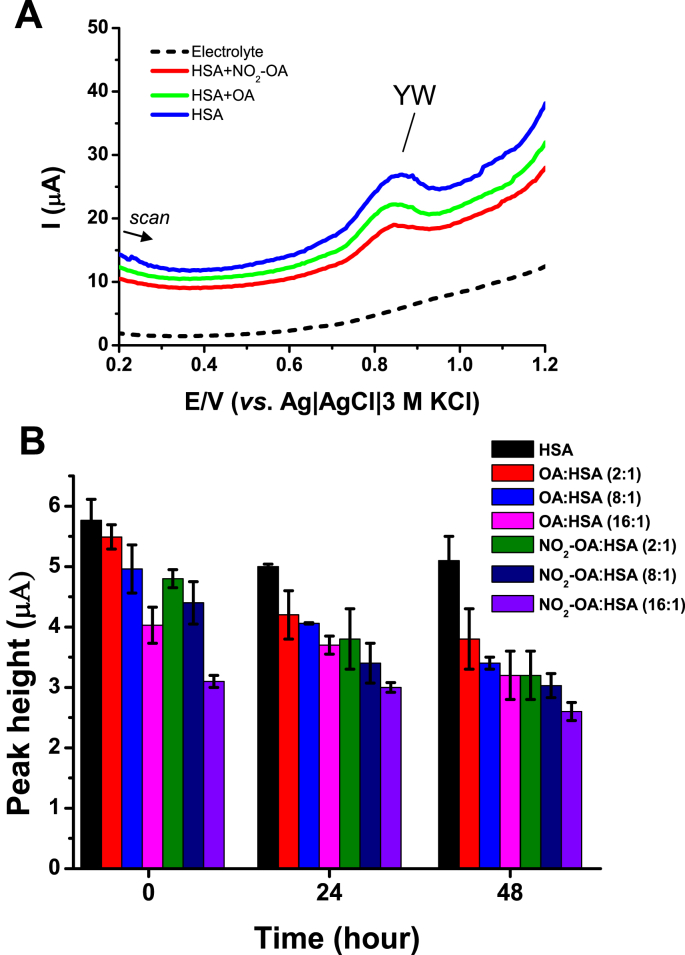
**(A)***Ex situ* SW voltammograms of HSA in absence or presence of nitro-oleic (NO_2_-OA) and oleic (OA) acid in acetate buffer (pH 5) in molar ratio 16:1 [FA]:[HSA] after 24 h of incubation. (**B)** Dependence of peak YW heights of HSA in absence or presence of fatty acids (FAs) in different molar ratios as indicated. HSA incubation with FAs was performed in 0.1 M phosphate buffer, pH 7.4, at 37 °C; the concentration of HSA in incubation mixtures was 6.25 μM and the FAs were added in the molar ratios: 2:1, 8:1, 16:1 [FA]:[HSA]. The incubation was performed for three different lengths of time: 0 h = the samples were analyzed immediately after HSA-FA mixing. SWV parameters: working electrode was PGE, accumulation time 30 s, initial potential: 0 V, end potential: +1.2 V, step potential: 5 mV, amplitude: 25 mV, frequency: 200 Hz. *Ex situ* analysis was performed as described in *Experimental* section.

In addition, an SWV procedure based on observing the HSA anodic peak YW also allowed characterization of HSA interaction with FAs (Fig. 6A). Peak YW corresponds to the oxidation of Tyr (Y) and Trp (W) residues in HSA (Fig. 5B). Similar to peak H, peak YW decreases after ligand binding [54,55]. A 48 h incubation of FAs with HSA resulted in HSA modification and a decrease in peak YW (Fig. 6B). A greater decrease was observed for NO_2_-OA than for OA. In addition to non-covalent association, NO_2_-OA adduction or the facilitation of the oxidation or nitration of HSA might be possible. To better understand this an LC-MS-based proteomic analysis of tryptic digests of HSA will be pursued in the future.

Finally, HSA samples incubated with NO_2_-OA were separated by SDS and native PAGE to evaluate aggregation or fragmentation processes. Based on SDS-PAGE analysis, we can postulate that there is no fragmentation of HSA after incubation with NO_2_-OA or OA (Fig. 4C). With native PAGE, no NO_2_-OA-induced aggregation of HSA was observed (Fig. 4D). In addition, significant changes in the electrophoretic mobility of HSA were apparent after NO_2_-OA association, in contrast to the HSA-OA control. The increase of electrophoretic mobility of HSA with increasing [NO_2_-OA]:[HSA] ratio is also apparent, Fig. 4E.

### 3.4 EPR evaluation of NO_2_-OA binding with HSA

**Fig. 7:**
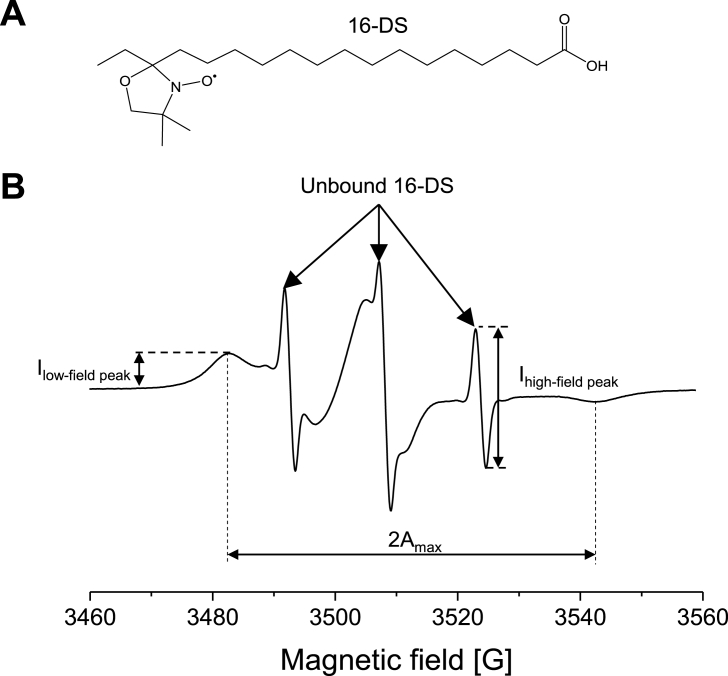
**(A)** Chemical structure of spin-probe 16-doxyl-stearic acid – a paramagnetic derivative of stearic acid containing an unpaired electron between the nitrogen and oxygen atom of the doxyl group. **(B)** EPR spectrum of HSA incubated with 16-DS at [16-DS]:[HSA] molar ratio 6:1. This spectrum consists of two main components: the broad anisotropic spectrum of 16-DS bound to HSA characterized by the spectrum width, 2A_max_, and the designated sharp triplet which corresponds to the unbound, freely tumbling 16-DS. The sample contained 0.1 mM HSA dissolved in 0.1 M phosphate buffer at pH 7.4.

To better understand HSA interactions with NO_2_-OA, EPR spectroscopy was used. Conformational changes of HSA and its interaction with various ligands have been widely studied *via* spin-probing/EPR methods [40,[56], [57], [58], [59], [60], [61], [62], [63], [64]]. This approach involves the incorporation of paramagnetic spin-probes into the proteins and detection of the signal arising from the paramagnetic moiety by EPR. Due to an ability to bind up to seven molecules of long-chain fatty acids [65,66], bovine and human serum albumin have been studied using paramagnetic derivatives of stearic acid [[56], [57], [58], [59], [60],[62], [63], [64]]. In this study, 16-doxyl-stearic acid (16-DS, Fig. 7A) was used to gain more insight into NO_2_-OA-HSA binding. 16-DS was first bound to HSA, and the 16-DS/HSA complex was further incubated with varying concentrations of NO_2_-OA or OA. This approach provides the opportunity to monitor the changes in the amount of both bound and unbound probe, originating from the presumed competition between 16-DS and NO_2_-OA or OA. Namely, a typical EPR spectrum of HSA complexed with 16-DS at a high [16-DS]:[HSA] molar ratio, 6:1, is composed of two main components (Fig. 7B), a broad anisotropic spectrum corresponding to the 16-DS bound to HSA, and an isotropic sharp triplet, arising from the freely tumbling 16-DS in aqueous solution [[56], [57], [58], [59], [60],[62], [63], [64]]. The amount of 16-DS bound to HSA is, in the first approximation (if the widths of peaks or whole spectra do not change significantly), proportional to the height of the low-field peak (I_low-field peak_), while the amplitude of the sharp high-field peak, I_high-field peak_ can provide information about the amount of unbound 16-DS [62,64]. Hence, changes in the amount of bound and unbound 16-DS can be monitored by observing the I_high-field peak_/I_low-field peak_ ratio. However, it should be emphasized that I_high-field peak_/I_low-field peak_ does not represent the ratio of the real amounts of unbound and bound 16-DS [62].

**Fig. 8:**
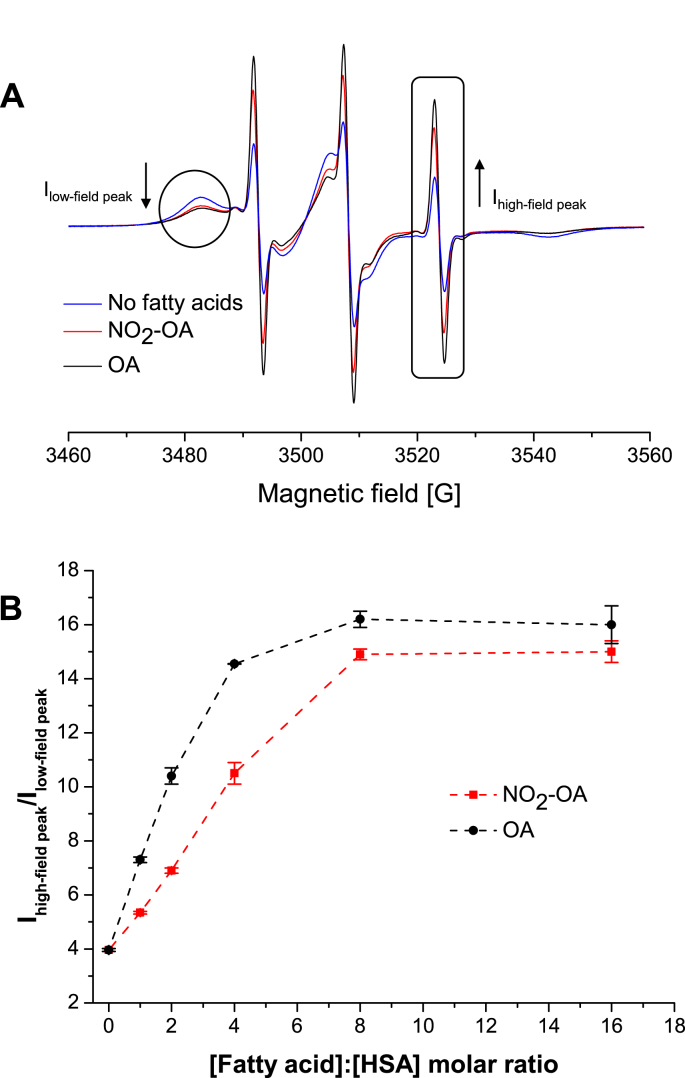
**(A)** EPR spectra of 16-DS/HSA complex in presence and absence of oleic (OA) and NO_2_-OA. The [FA]:[HSA] molar ratio in these samples was 4:1. **(B)** Dependence of I_high-field__peak_/I_low-field peak_ ratio measured from EPR spectra of 16-DS bound to HSA on [FAs]:[HSA] molar ratio. All samples used for obtaining data presented in panels **A** and **B** contained 0.1 mM HSA (dissolved in 0.1 M phosphate buffer, pH 7.4) and 0.6 mM 16-DS. Unlabeled fatty acids were incubated with 16-DS/HSA complex at 37 °C for 30 min. Afterwards, the samples were cooled down to room temperature, and subsequently EPR spectra were acquired.

From the EPR spectra obtained for different [FA]:[HSA] molar ratios, the addition of NO_2_-OA or OA induced an increase in the I_high-field peak_ and a decrease in I_low-field peak_ (Fig. 8A). The dependence of I_high-field peak_/I_low-field peak_ on the increasing [FA]:[HSA] molar ratio for both NO_2_-OA and OA is given in Fig. 8B. The increase in I_high-field peak_/I_low-field peak_ values with the rising [FA]:[HSA] molar ratio obtained for NO_2_-OA and OA indicates that these two fatty acids are able to displace 16-DS previously bound to HSA. Furthermore, it is evident that up to a [FA]:[HSA] molar ratio of ∼8:1, each new additional molecule of NO_2_-OA/OA leads to an increase in the amount of unbound 16-DS. The curve shows a saturation at molar ratios greater than ∼8:1, consistent with the existence of seven long-chain FA binding sites [40,65]. Also, the data presented in Fig. 8B suggest that OA displaces the 16-DS molecules from HSA to a greater extent than NO_2_-OA. Multiple studies have shown that unlabeled medium- and long-chain fatty acids [58,59,64], and more specifically OA [63], compete with various spin-labeled fatty acids (SLFAs) upon binding to serum albumin (HSA and BSA), thereby diminishing the amount of the bound SLFA. From these data it was concluded that SLFAs bind to the same binding sites as the unlabeled FAs. All of these findings are consistent with the results reported in this study. It may be concluded that up to 7 molecules of NO_2_-OA bind to HSA, presumably to the well-known and characterized FA binding sites designated FA1-FA7 [40,65]. The OA binding sites are highlighted in the surface and ribbon models in Fig. 5B and Fig. S5 in Supplementary Information.

### 3.5 NO release and stability of NO_2_-OA

**Fig. 9:**
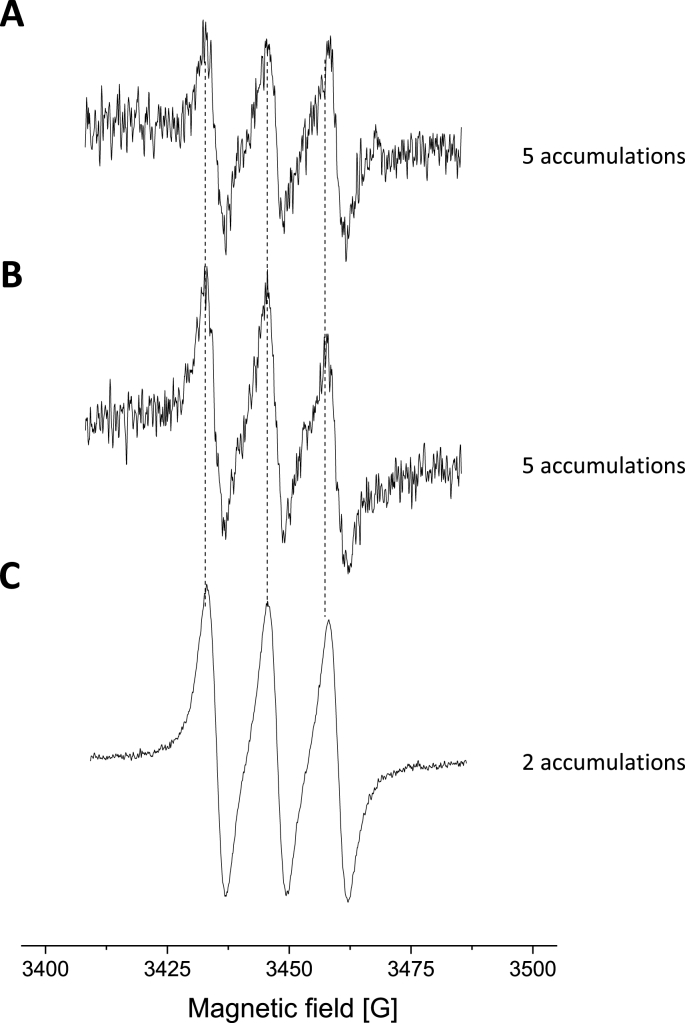
EPR spectra of NO radicals trapped by Fe(DTCS)_2_. The NO radical was released from: **(A)** NO_2_-OA dissolved in 0.1 M phosphate buffer, pH 7.4, **(B)** NO_2_-OA dissolved in deionized water and **(C)** chemical NO-generator, sodium-nitroprusside (SNP).

EPR spectroscopy, using Fe(DTCS)_2_ as the spin-trapping agent, was employed to detect whether NO_2_-OA generated NO radicals (Fig. 9). By comparing the EPR signals obtained for NO_2_-OA and sodium-nitroprusside (SNP), an NO donor, it was observed that NO_2_-OA generates NO radicals in buffer solution (or water). The EPR signal of the NO-Fe(DTCS)_2_ complex is lower in a pH 7.4 environment because of the oxidation of Fe^2+^ into Fe^3+^, which is needed to form a stable spin-trapping complex, see Fig. 9.

**Fig. 10:**
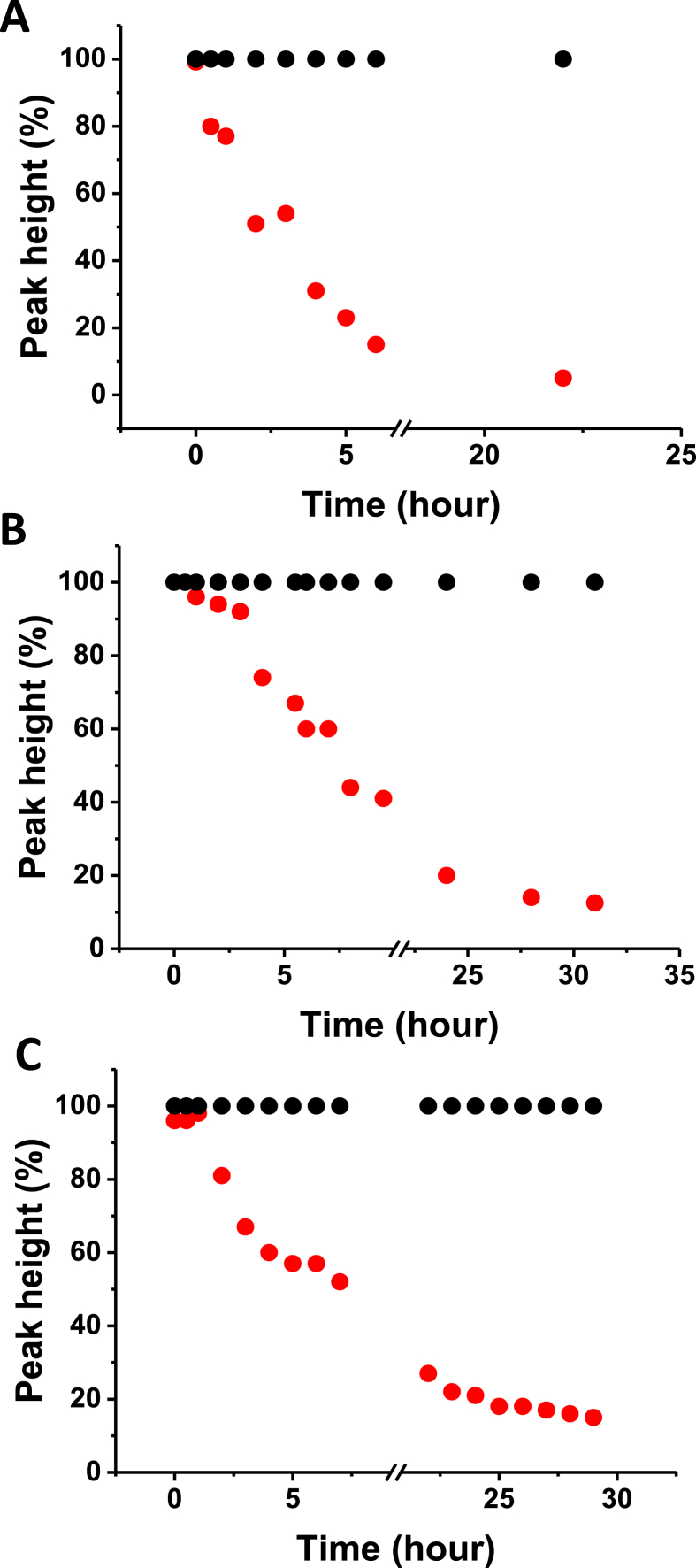
Time stability of 8 μM NO_2_-OA monitored *via* a decrease in CPS peak NO. CPS analysis of NO_2_-OA stability was performed directly in supporting electrolyte at **(A)** pH 5, **(B)** 7.4 and **(C)** 9; time of accumulation 30 s at open current circuit was used, *I*_str_ −35 μA. Black dots = control = newly prepared (fresh) NO_2_-OA solution at concentration of 8 μM. For more details, see Fig. 2A.

For monitoring the stability of NO_2_-OA, the electrochemical peak NO was observed over time using CPSA (Fig. 10). The stability of NO_2_-OA was studied in phosphate or Britton-Robinson buffer at pH 5; 7.4 and 9 for at least 24 h. A freshly prepared methanolic stock solution of NO_2_-OA solution was used as a control. During the first 2 h, the NO_2_-OA is quite stable under experimental conditions used. From the point of view of long-term stability, an approx. 80% decrease in NO_2_-OA was observed at pH 7.4 after 24 h. The pilot stability data reported here should be investigated further in more detail. The reported data should be taken into account in further biochemical or pharmacological studies conducted in aqueous buffered media.

### 3.6 Biological consequences and further prospects

With regard to the biophysical, cell signaling and pharmacological properties of NO_2_-OA, the present results add important new perspective by documenting that NO_2_-OA interacts with biological targets *via* both covalent and non-covalent mechanisms. Covalent reactions with protein and low molecular weight thiols and are central to both the transduction and cessation of NO_2_-OA signaling. This was demonstrated *in vivo* where nitro-alkenes facilitate the post-translational modification of kinetically-privileged thiols of key target proteins of high biological relevance as described above in detail, (see Introduction). In the context of gene expression regulation, the alkylation of Cys residues in transcriptional regulatory proteins such as the Keap1 regulator of Nrf2 signaling, PPARγ and NF-kappaB account for important aspects of NO_2_-OA signaling *in vivo* [5,18,24]. This is demonstrated in diverse cell and murine models of cancer, and metabolic syndrome and cardiopulmonary and renal inflammatory responses [19,[67], [68], [69]]. Gene expression response studies in human vascular cells reinforce that there is extensive pleiotropic modulation of adaptive cell responses that are related to cell proliferation, lipid metabolism, antioxidant and anti-inflammatory reactions [70,71]. The present study also shows the important role that plasma proteins can play in non-covalent systemic transport of NO_2_-OA, since the large pool of plasma albumin has the capacity of binding and stabilizing extensive quantities of this and other electrophilic fatty acids. While we appreciate that esterification of NO_2_-OA into complex lipids such as triglycerides also contributes to the stabilization and transport of NO_2_-OA [72], more work is needed to fully understand the absorption, storage and transport of nitrated fatty acids that are orally consumed or formed during digestion.

## 4 Conclusions

In this report, we focused on evaluating the redox behavior of NO_2_-OA and its ability to bind to HSA. The nitro group in NO_2_-OA undergoes electrochemical reduction at around −0.75 V at neutral pH. Based on observations of this reduction process, we can quantitatively monitor the reactivity of NO_2_-OA, using OA as the negative control. Using this electrochemical approach, NO_2_-OA stability was monitored. During the first 2 h NO_2_-OA is quite stable, whereas an approx. 80% decrease in NO_2_-OA level was observed at pH 7.4 in an aqueous environment after 24 h. The above electrochemical data were supported by computational quantum mechanical modeling. The DFT calculations indicated that both the C9 and C10 NO_2_-positional isomers of NO_2_-OA occurred in two conformers with different internal angles (69° and 110°) between both the methyl and carboxylate ends. Both regioisomers have LUMO energies of around −0.7 eV, consistent with the electrophilic character of fatty acid nitroalkenes. Knowledge of the internal angles of 9- and 10-NO_2_-OA is useful for modeling NO_2_-OA interactions with nucleophilic amino acids its many biological target proteins. The binding of NO_2_-OA with HSA was non-covalent and displayed a similar stoichiometry to OA, *i.e.* 7:1 [FA]:[HSA]. The binding experiments were performed using electrochemistry, native electrophoresis and electron paramagnetic resonance (EPR) spectroscopy using 16-doxyl stearic acid. In addition to non-covalent association, NO_2_-OA adduction or the facilitation of the oxidation or nitration of HSA might be possible as confirmed using SWV approach. EPR experiments also showed that NO-release in NO_2_-OA is possible and ultimately yielded an NO radical, detectable *via* an Fe(DTCS)_2_ spin-trap. This observation is consistent with previous reports, that also show that in more complex biological milieu (plasma, membranes, micelles, complex lipids) that NO release is not apparent [29,72,73].

The data presented here add to our understanding of the reactivities and plasma protein interactions of NO_2_-OA and can be useful for further molecular studies focusing on natural and non-natural lipid electrophiles [74] of biological and pharmacological relevance.
