# Supplementary material for: Redox properties and human serum albumin binding of nitro-oleic acid
Source: Redox Biol. 2019 May 8;24:101213. doi: 10.1016/j.redox.2019.101213 (PMC6554544; doi:10.1016/j.redox.2019.101213)

Supporting Information:

Redox Properties and Human Serum Albumin Binding of Nitro-Oleic Acid

Martina Zatloukalova,[a] Milos Mojovic,[b] Aleksandra Pavicevic,[b] Martin Kabelac,[c] Bruce A. Freeman,[d] Michaela Pekarova[e] and Jan Vacek[a,e*]

[a] Department of Medical Chemistry and Biochemistry, Faculty of Medicine and Dentistry, Palacky University, Hnevotinska 3, Olomouc 775 15, Czech Republic

[b] Faculty of Physical Chemistry, University of Belgrade, Studentski trg 12-16, Belgrade, Serbia

[c] Department of Chemistry, Faculty of Science, University of South Bohemia, Branisovska 31, Ceske Budejovice 370 05, Czech Republic

[d] Department of Pharmacology and Chemical Biology, University of Pittsburgh School of Medicine, Pittsburgh, PA, 15261, USA

[e] The Czech Academy of Sciences, Institute of Biophysics, Kralovopolska 135, Brno 612 65, Czech Republic

[*]) corresponding author: J. Vacek, e-mail: jan.vacek@upol.cz; tel.: +420-585-632-303

Table S1. Energy differences (in kJ/mol) between 'open' and 'closed' conformation. The 'closed' one is more stable.

Oleic acid (o)	0.73	9-Nitro-oleic acid (o)	0.02	10-Nitro-oleic acid (o)	0.95
Oleic acid (m)	0.78	9-Nitro-oleic acid (m)	0.02	10-Nitro-oleic acid (m)	1.00
Oleic acid (w)	0.80	9-Nitro-oleic acid (w)	0.02	10-Nitro-oleic acid (w)	1.02

Abbrev: (o) in *n*-octanol, (m) in methanol, (w) in water

Table S2. Energies of HOMO, LUMO and its differences (in eV) obtained at DFT level with B3LYP functional and 6-311++G(d, p) basis set.

	HOMO	LUMO	Delta (eV)
open conformation			
Oleic acid (o)	-7.802	1.312	9.114
Oleic acid (m)	-8.183	1.245	9.429
Oleic acid (w)	-8.281	1.226	9.508
9-Nitro-oleic acid (o)	-7.807	-0.636	7.170
9-Nitro-oleic acid (m)	-8.187	-0.746	7.441
9-Nitro-oleic acid (w)	-8.285	-0.774	7.511
10-Nitro-oleic acid (o)	-7.815	-0.640	7.175
10-Nitro-oleic acid (m)	-8.189	-0.744	7.445
10-Nitro-oleic acid (w)	-8.285	-0.771	7.515
closed conformation			
Oleic acid (o)	-7.802	1.296	9.098
Oleic acid (m)	-8.183	1.207	9.390
Oleic acid (w)	-8.281	1.182	9.464
9-Nitro-oleic acid (o)	-7.808	-0.621	7.187
9-Nitro-oleic acid (m)	-8.187	-0.745	7.442
9-Nitro-oleic acid (w)	-8.285	-0.774	7.511
10-Nitro-oleic acid (o)	-7.816	-0.627	7.189
10-Nitro-oleic acid (m)	-8.190	-0.731	7.459
10-Nitro-oleic acid (w)	-8.286	-0.758	7.529

Abbrev: (o) in *n*-octanol, (m) in methanol, (w) in water

Fig. S1. (A) CV records of 5 µM NO_2-OA in Britton-Robinson buffer at various pH. **(B)** Dependence of peak NO potential on pH of supporting electrolyte (Britton-Robinson buffer). CV conditions: working electrode PGE, start potential 0 V, first vertex potential –1.85 V, second vertex potential +1 V, step potential 5 mV, scan rate 1 V/s, adsorption time: 30 s. Electrolyte (black line) was measured at pH 3.

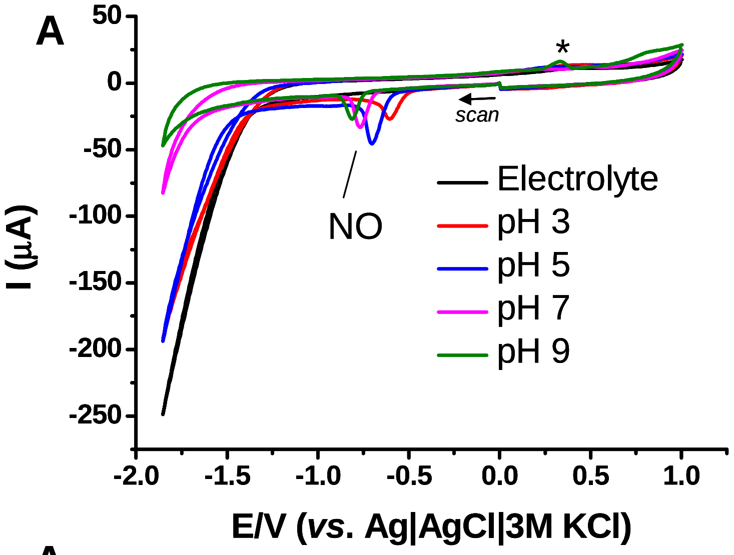

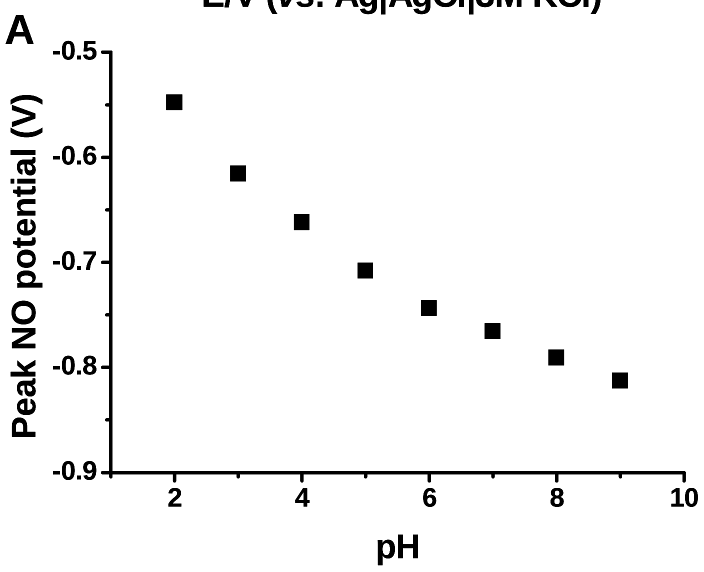

Fig. S2. (A) Cyclic voltammograms of 20 µM NO_2-OA in 0.1 M phosphate buffer at pH 7.4 at various scan rates (v). CV conditions: start potential –0.25 V, vertex potential –1.25 V, step potential 5 mV. **(B)** Dependence of peak NO height on scan rate (v) expressed as $v^{1/2}$.

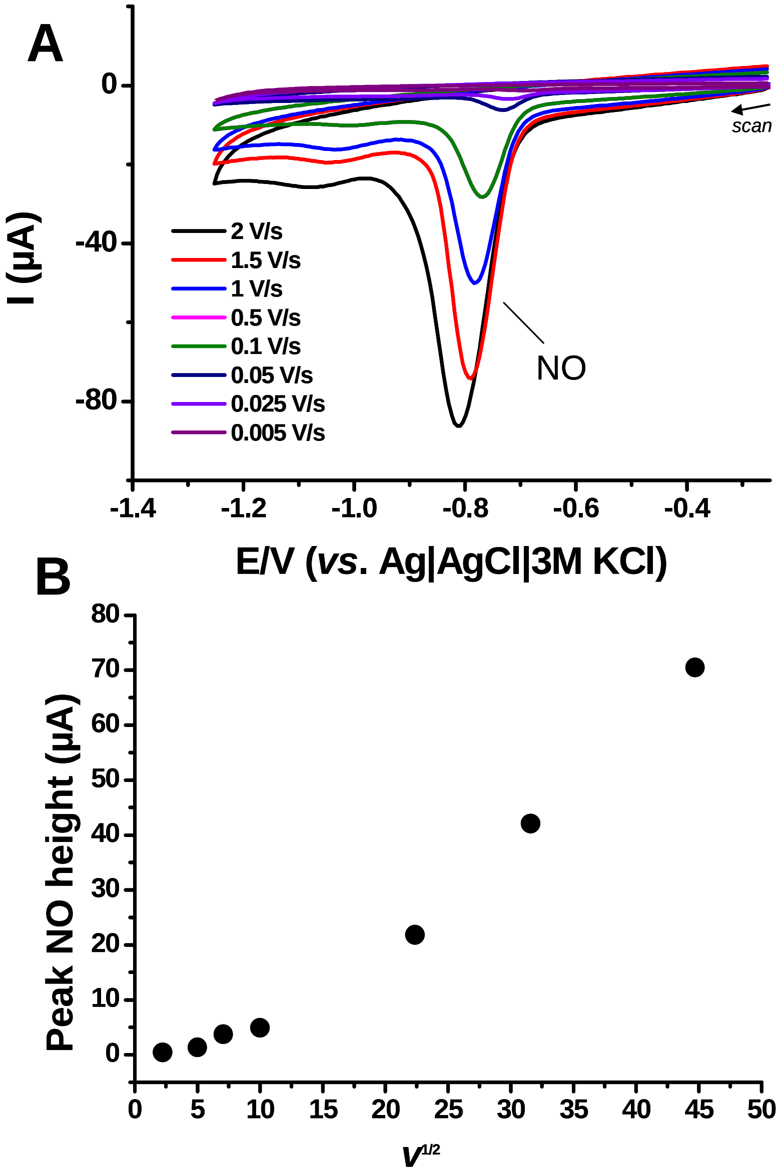

Fig. S3. (A) Dependence of SWV peak NO of 8 µM NO_2-OA on accumulation time measured using pyrolytic graphite electrode in 0.1 M phosphate buffer at pH 7.4. For more details, see figure **1C** in the main text. *Inset*: Selected SWV records related to panel **A**. **(B)** CPS analyses of racemic mixture 9,10-NO_2-OA (8 µM) and pure isomers: 9-NO_2-OA and 10-NO_2-OA (10 µM for both) in 0.1 M phosphate buffer (pH 6.5); I_{str}= –35 µA. For more details, see figure **2A** in the main text.

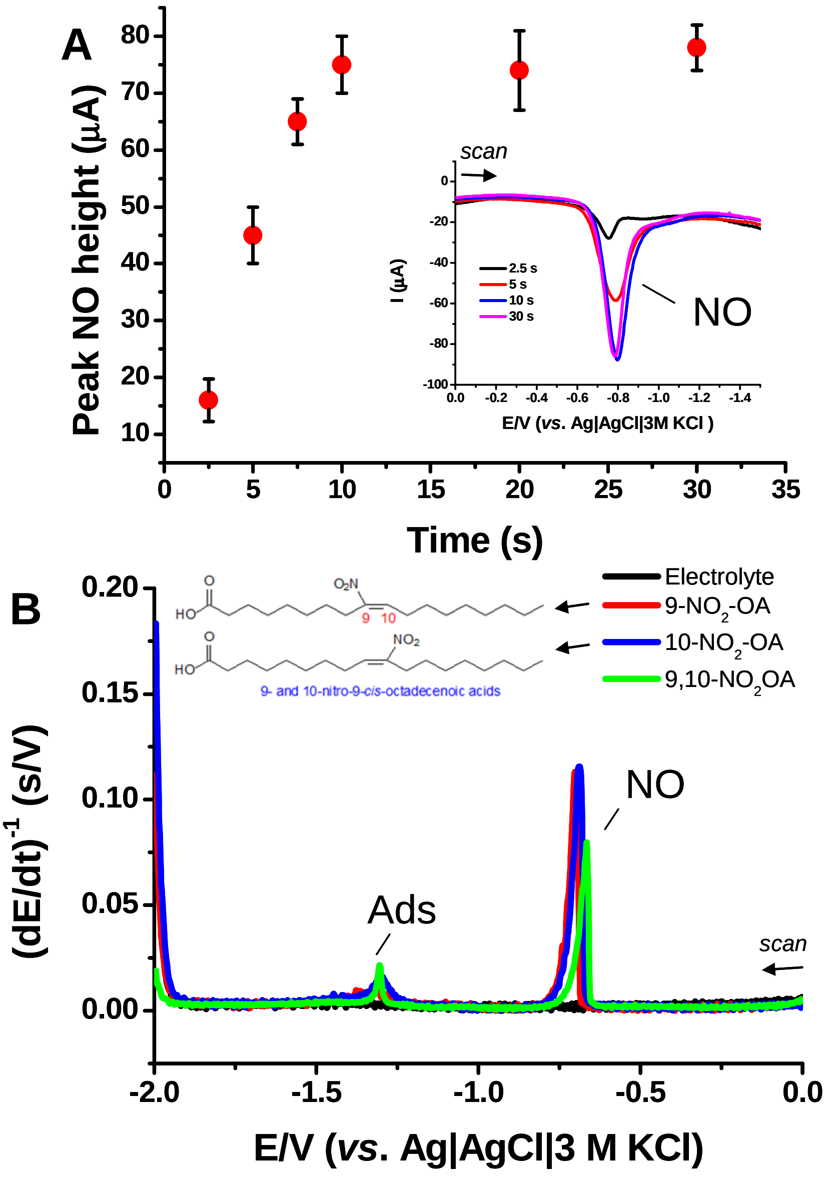

Fig. S4. Cyclic voltammograms of 20 µM NO_2-OA and OA in Briton-Robinson buffer at pH 5. CV parameters: working electrode was PGE, start potential 0 V, first vertex potential –1.85 V (black and dashed grey line), –1.1 V (red line) and –0.5 V (blue line), second vertex potential +1 V, step potential 5 mV, scan rate 1 V/s.

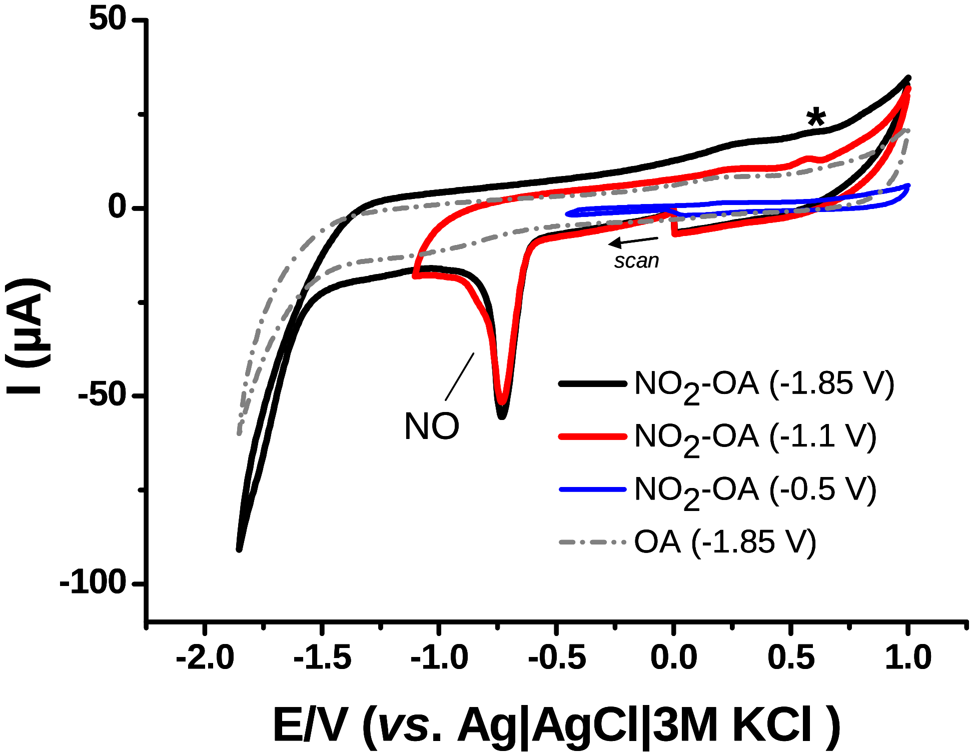

Fig. S5. Ribbon models of HSA (PDB code: 1GNI) with electroactive amino acid residues highlighted. **(A)** Cys – red, His – blue, Arg – yellow, Lys – magenta. **(B)** Tyr – cyan, Trp – brown, oleic acid (OA) – black. The left and right images are mutually rotated by 180° along the vertical axis for each panel.

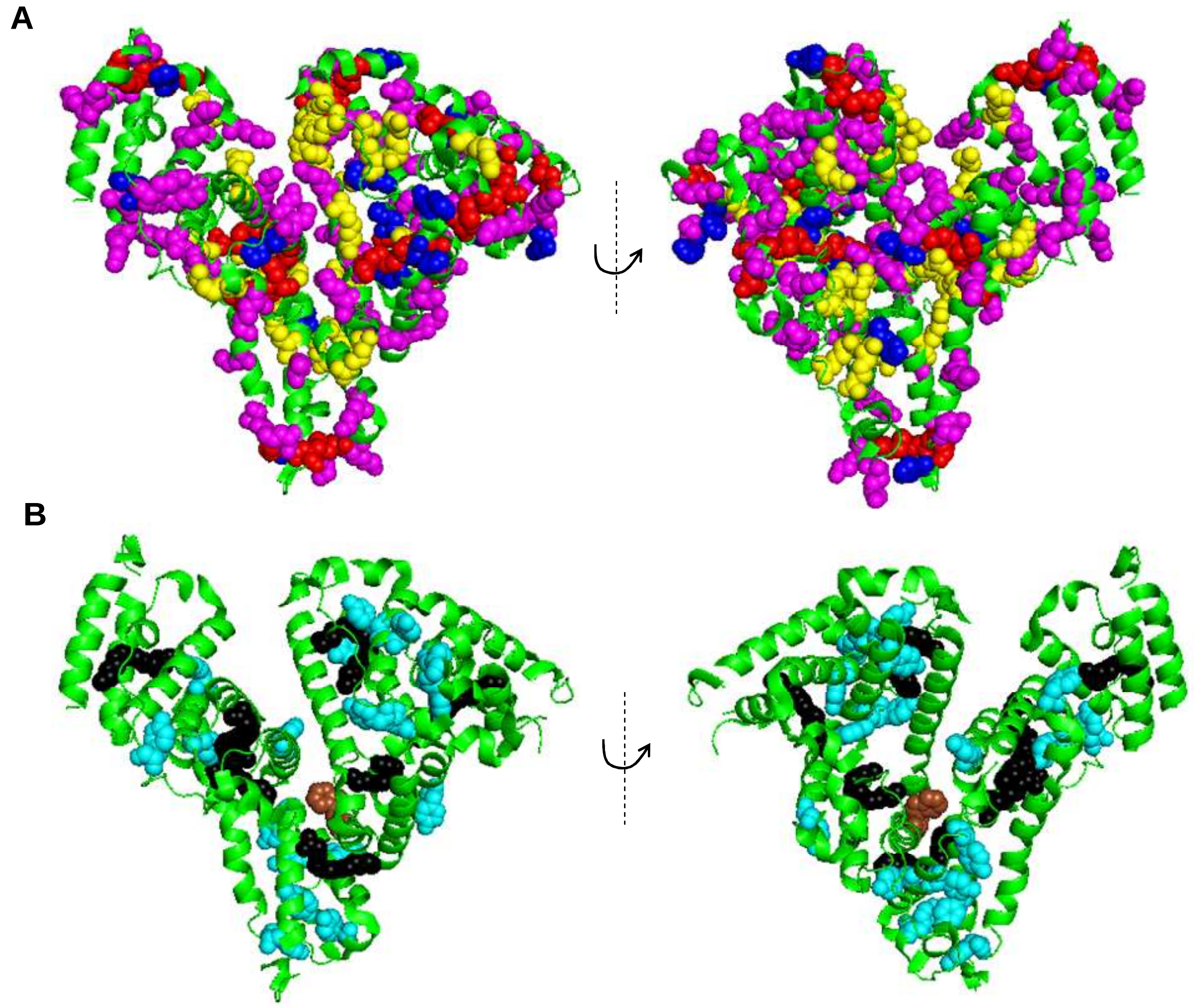

Supplement: Multimedia component 1 [file mmc1.pdf]
